# Interventricular septal hydatid cyst of the heart presenting with syncope: a rare case report

**DOI:** 10.1093/ehjcr/ytag525

**Published:** 2026-07-30

**Authors:** Abdul Wahed Sidiqi, Khaiber Sidiqi, Asma Rahbeen, Hedayatullah Lalzai, Manizha Noorandish

**Affiliations:** Department of Cardiology, Faculty of Medicine, Kabul University of Medical Sciences, Abu Ali Ibn Sina, Ata Turk Avenue, Jamal Mena, District 3, Kabul 1001, Afghanistan; Department of Cardiology, Ariana Medical Complex, Red Crescent Compound, District 5, Afshar Area, Kabul 100535, Afghanistan; Department of Cardiovascular Surgery, Ariana Medical Complex, Red Crescent Compound, District 5, Afshar Area, Kabul 100535, Afghanistan; Department of Cardiology, Ariana Medical Complex, Red Crescent Compound, District 5, Afshar Area, Kabul 100535, Afghanistan; Department of Ultrasound and Doppler, Ariana Medical Complex, Red Crescent Compound, District 5, Afshar Area, Kabul 100535, Afghanistan; Department of Cardiology, Ariana Medical Complex, Red Crescent Compound, District 5, Afshar Area, Kabul 100535, Afghanistan

**Keywords:** Interventricular septum hydatid cyst, Cardiac echinococcosis, Exertional syncope, Echocardiography, RVOT obstruction, Case report, Serologic test

## Abstract

**Background:**

Cardiac echinococcosis is scarce, constituting <2% of all hydatid cyst infections. Isolated cardiac involvement is infrequent, often affecting the left ventricle. The interventricular septum is an even rarer location, with limited cases reported in the literature.

**Case summary:**

A 50-year-old woman presented with a history of chest discomfort, fatigue, exertional dyspnoea, and recurrent syncope. Transthoracic and transoesophageal echocardiography revealed a well-defined, large, intramyocardial cystic mass within the interventricular septum, protruding into the right ventricular cavity and outflow tract (RVOT). Doppler imaging demonstrated turbulent flow with RVOT stenosis (peak/mean gradients: 95/46 mmHg at rest and 107/67 mmHg at exercise, respectively). The serologic Cassoni test confirmed echinococcal infection, albendazole therapy was initiated, and surgical resection was performed successfully. A 2-year follow-up showed no recurrence.

**Discussion:**

This case highlights the unusual presentation of an isolated interventricular septal hydatid cyst, which manifested with exertional syncope. It demonstrates the crucial role of early diagnosis via echocardiography, especially in resource-limited settings, and serologic testing to prevent potentially fatal complications. It underscores the need for a multidisciplinary approach combining surgical excision and anti-parasitic therapy to achieve optimal outcomes in endemic settings.

Learning points

- Reports a rare isolated interventricular septal hydatid cyst causing recurrent syncope due to right ventricular cavity and outflow tract obstruction.
- Demonstrates the critical role of echocardiography in the early detection of life-threatening intracardiac cysts in resource-limited endemic regions.
- Highlights the value of multidisciplinary management, combining surgical excision with perioperative albendazole, to prevent catastrophic complications.

## Introduction

Echinococcosis is an infectious disease of parasitic and zoonotic nature, induced by species of the genus *Echinococcus*, including *Echinococcus granulosus*, *Echinococcus multilocularis*, and *Echinococcus vogeli*. Hydatid disease is endemic in regions such as South America, the Middle East, Africa, and Central Asia, commonly affecting the liver and lungs, whereas cardiac involvement is rare.^1^

Cardiac echinococcosis, though rare, poses substantial diagnostic and therapeutic challenges. The prevalence of cardiac hydatid cysts ranges from 0.01% to 2%,^2^ with the interventricular septum being an uncommon site.^3^ Cardiac hydatid cysts remain silent in 90% of cases. The disease may lead to life-threatening complications, including heart failure, pulmonary embolism, conduction disturbances and arrhythmias, valve obstruction, pericardial effusion, and sudden cardiac death.^4^

Echocardiography plays an integral role in the initial screening and dynamic evaluation of patients with cardiac hydatid disease,^5^ while computed tomography (CT) and magnetic resonance imaging (MRI) are complementary modalities.^6^ Serological assays such as enzyme-linked immunosorbent assay (ELISA) and indirect hemagglutination (IHA) provide greater sensitivity and specificity than the traditional Cassoni test for diagnosis of hydatid cyst.^7^

This case report describes a rare instance of an isolated interventricular septal hydatid cyst resulting in right ventricular outflow tract (RVOT) obstruction and syncope, highlighting the necessity of early diagnosis to prevent life-threatening complications. The report also demonstrates the effectiveness of a combined surgical and adjunctive pharmacological strategy in optimizing patient outcomes, especially in resource-limited, endemic regions such as Afghanistan.

## Summary figure

**Figure:**
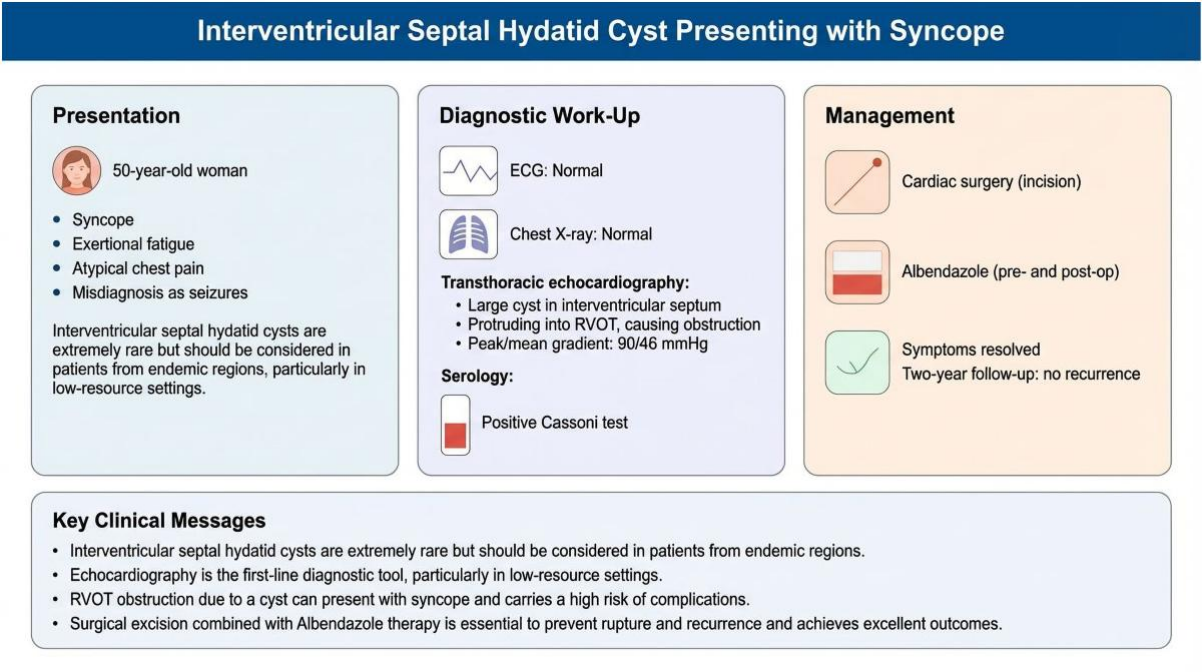


## Case presentation

A 50-year-old woman who lived in a rural area, with no prior cardiac history, presented to the Ariana Medical Complex with a history of chest discomfort, fatigue, exertional dyspnoea, and recurrent syncope; she has denied any history of fever, palpitations, or recent travel. Physical examination revealed a Grade III systolic ejection murmur at the left upper sternal border, suggestive of RVOT obstruction. Heart sounds were normal, with preserved S1 and S2 and no gallops. There was no cyanosis, clubbing, or peripheral oedema. Vital signs were stable, with no clinical evidence of haemodynamic disturbance. Before referral to this tertiary healthcare centre for further evaluation, her syncopal episodes were presumed to be seizure-related, resulting in several months of ineffective antiepileptic therapy.

### Diagnostic work-up

**Figure 1:**
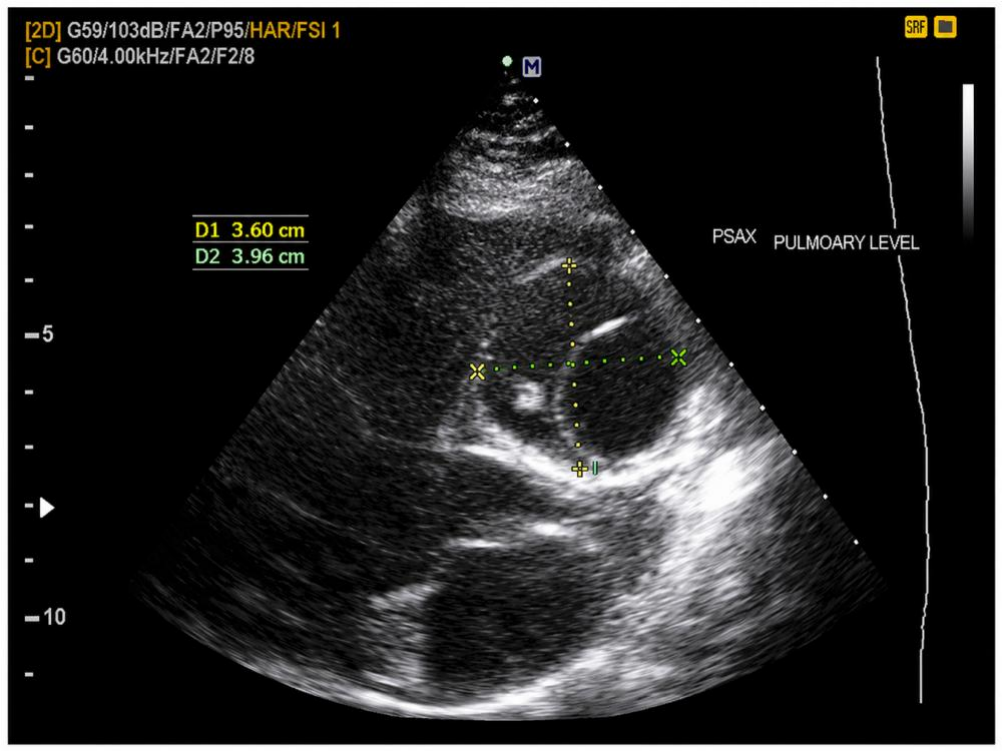
Transthoracic echocardiography, parasternal short-axis view at the pulmonary level, demonstrating a well-defined 3.6 × 3.9 cm intramyocardial cystic mass within the interventricular septum protruding towards the right ventricular cavity.

**Figure 2:**
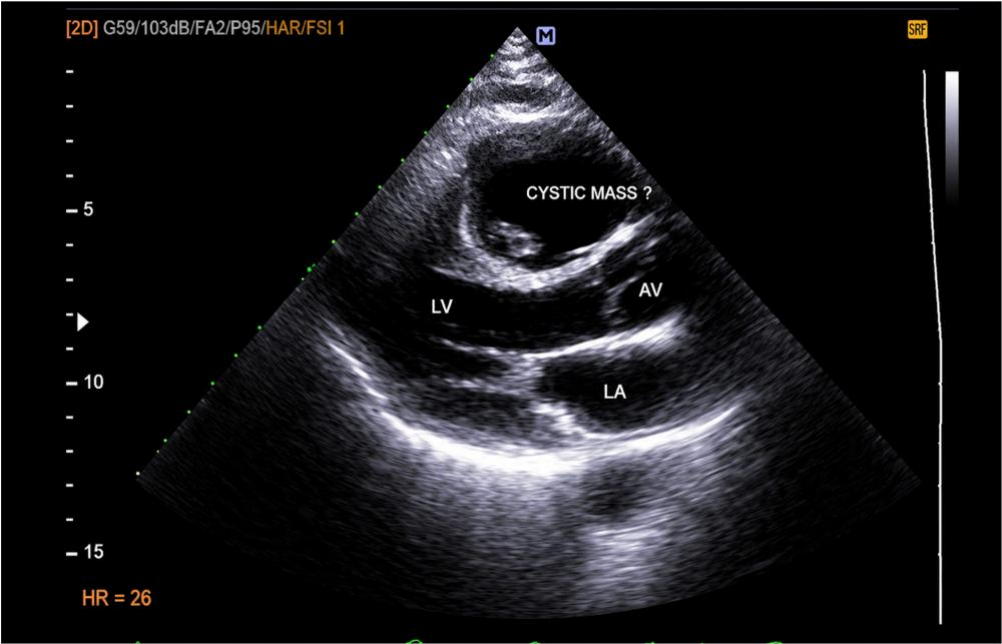
Transthoracic echocardiography, parasternal long-axis view, demonstrating a large intramyocardial cystic mass within the interventricular septum protruding into the right ventricular outflow tract, causing dynamic right ventricular outflow tract obstruction with preserved left ventricular systolic function.

**Figure 3:**
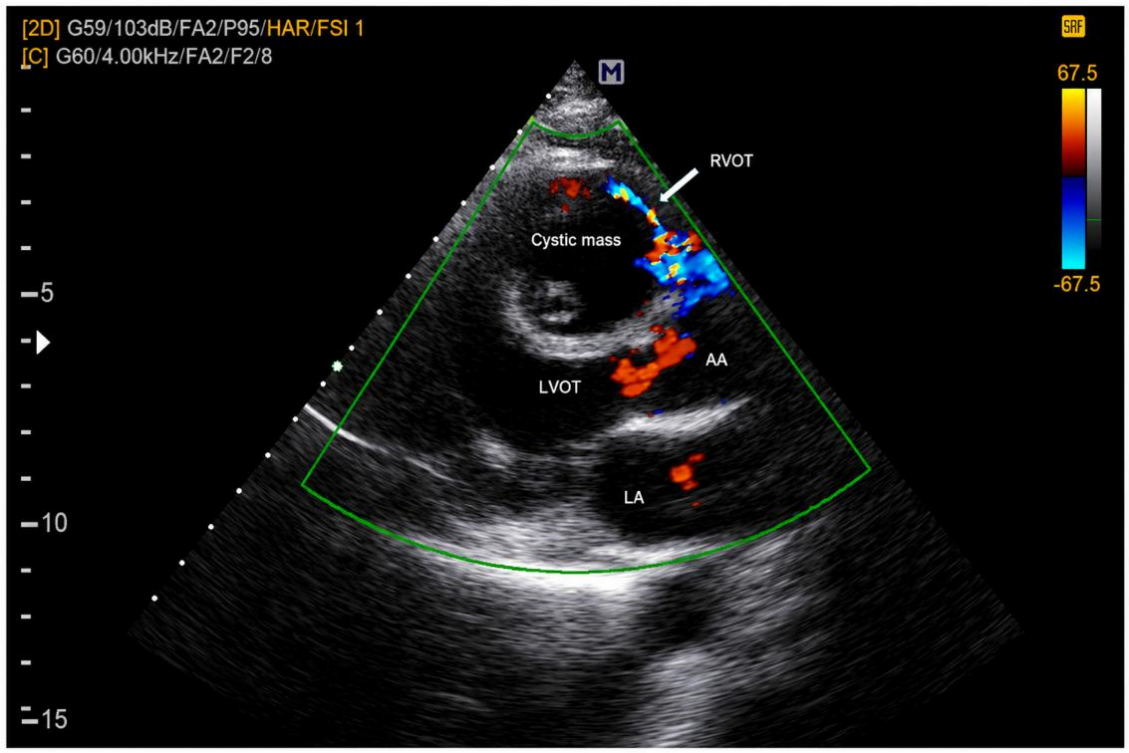
Colour Doppler transthoracic echocardiography (parasternal long-axis view) demonstrating turbulent flow across the right ventricular outflow tract secondary to protrusion of the interventricular septal hydatid cyst, consistent with severe right ventricular outflow tract obstruction.

**Figure 4:**
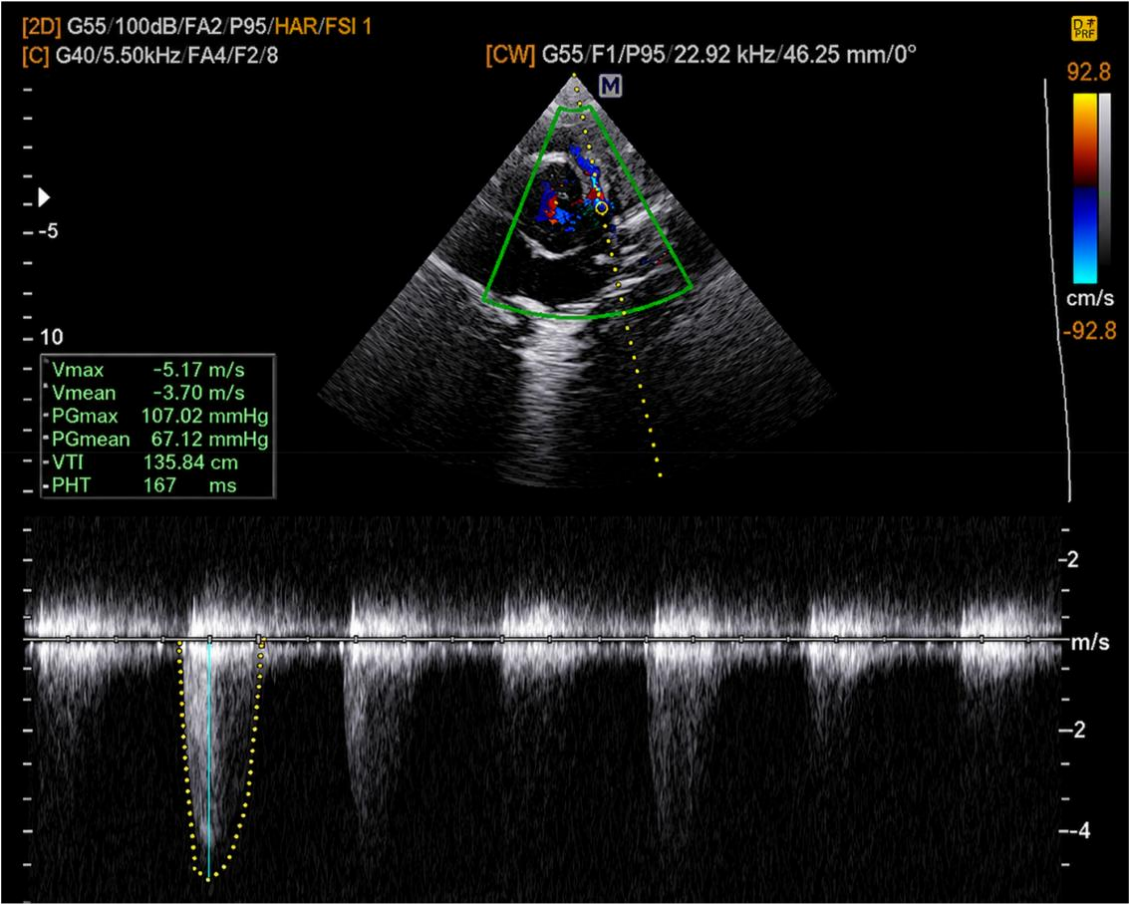
Continuous-wave Doppler echocardiography across the right ventricular outflow tract, obtained during exercise (stress echocardiography), demonstrates a high-velocity systolic jet (*V*max 5.17 m/s) with peak and mean pressure gradients of 107 and 67 mmHg, respectively, confirming severe right ventricular outflow tract obstruction secondary to an interventricular septal hydatid cyst mechanical effect.

Continuous electrocardiogram (ECG) monitoring during the hospitalization showed a normal sinus rhythm, while chest radiography and abdominal ultrasound revealed no abnormalities. Transthoracic echocardiography (TTE) showed a large (3.6 × 3.9 cm) intramyocardial cystic mass (*Figure 1*) within the interventricular septum protruding into the RVOT (*Figure 2*). Colour Doppler imaging revealed turbulent flow in the RVOT (*Figure 3*), with peak and mean gradients of 107/67 mmHg during exercise (*Figure 4*) and 95/46 mmHg at rest, suggesting severe RVOT obstruction. There was normal biventricular systolic function, with no evidence of left ventricular outflow tract obstruction.

**Figure 5:**
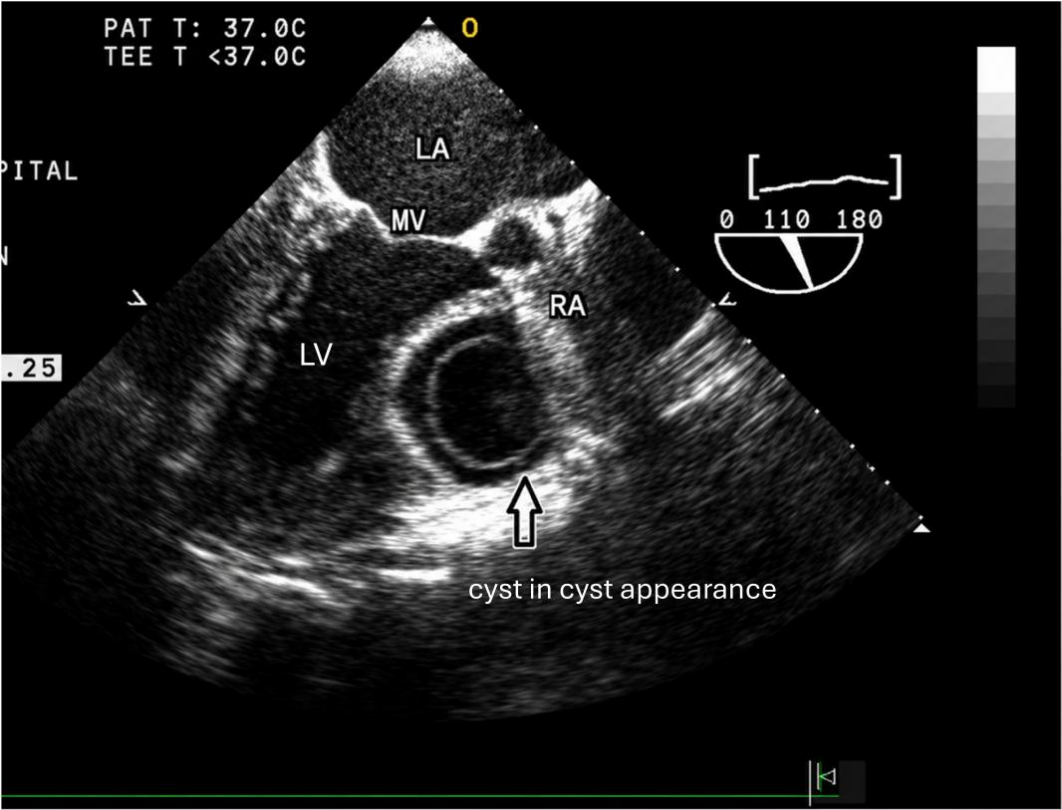
Transoesophageal echocardiography, mid-oesophageal view, demonstrating a well-defined intramyocardial cystic lesion within the interventricular septum (arrow) with a characteristic ‘cyst-in-cyst’ appearance, protruding towards the right ventricular cavity consistent with cardiac hydatid disease.

Transoesophageal echocardiography (TEE) demonstrated a well-defined intramyocardial cystic mass with characteristic ‘cyst-in-cyst’ appearance (*Figure 5*). As more sensitive serological assays, such as ELISA and IHA, were unavailable at the time of diagnosis, the Cassoni test was performed, yielding a positive result. There was no evidence of hepatic, pulmonary, or systemic involvement on further imaging. The absence of cardiac CT and MRI represents a limitation of this case due to resource constraints; therefore, the diagnostic workup relied on echocardiography and serological testing.

### Management and outcome

Pharmacologic therapy with albendazole was administered at 400 mg orally twice daily, initiated 1 month preoperatively to reduce cyst viability. As the patient was symptomatic, to prevent the risk of rupture, embolization, and conduction disturbances, the patient underwent open-heart surgery under cardiopulmonary bypass; the cyst was punctured, aspirated, and irrigated with a scolicidal agent, and the germinative membrane was completely enucleated from the interventricular septum, followed by capitonnage of the residual cavity. Histopathology confirmed a hydatid cyst with laminated (ectocyst) and germinative (endocyst) layers and multiple daughter cysts with no viable protoscoleces detected post-irrigation.

After the convalescent period, the patient’s symptoms had totally subsided. Post-operative echocardiography revealed post-surgical sequelae in the intraventricular septum, but the RVOT peak/mean gradient was within the normal range (25/8 mmHg). The patient continued treatment with albendazole for 6 months post-surgery to prevent recurrence and to eliminate any residual infection. Serial echocardiographic monitoring over a two-year period revealed no evidence of recurrence.

## Discussion

*Echinococcus granulosus* is a cestode (tapeworm), classified as a parasitic helminth within the phylum *Platyhelminthes*.^8^ Human act as aberrant intermediate host and become infected via the faecal–oral route by ingesting eggs from contaminated food, water, or through direct contact with definitive hosts (typically dogs). Once ingested, the eggs hatch in the small intestine, releasing oncospheres that penetrate the intestinal mucosa. These oncospheres enter the portal circulation, where the majority are filtered by the liver (50–70%) or lungs (20–30%). Cardiac involvement (<2%) occurs when oncospheres bypass these primary capillary beds and reach the myocardium via the coronary circulation.^8–11^

The dynamic mechanical environment of the myocardium is thought to limit echinococcal larval sequestration, cyst implantation, and subsequent intramyocardial expansion, thereby contributing to the exceptional rarity of primary cardiac echinococcosis,^2,3^ The left ventricle is the most frequently involved site (75%), followed by the right ventricle (18%). Less commonly affected areas include the pericardium (10%), left atrium (8%), right atrium (4%), and the interventricular septum (4%).^3^

In our case, the patient presented with a large, isolated interventricular septal cyst protruding into the RVOT, which demonstrates a rare localization of the cyst. While cardiac hydatid cysts may remain asymptomatic for years, clinical manifestations typically emerge from mass effect, outflow tract obstruction, or complications such as rupture and embolization.^12^

Our patient’s clinical presentation, specifically syncope in the context of an interventricular septal cyst, is thought to be haemodynamically driven; as the cyst expands, it causes severe, dynamic RVOT obstruction, leading to a critical reduction in cardiac output and subsequent cerebral hypoperfusion during exertion.^13^ However, septal cysts have a unique propensity to disrupt the cardiac conduction system, potentially precipitating complete heart block or paroxysmal arrhythmias, which must be considered in the differential diagnosis of syncope in endemic regions.^14^ The absence of significant arrhythmia in this case suggests that mechanical mass effect may serve as a potential mechanism for syncopal attacks, rather than arrhythmias.

In this case, despite severe RVOT obstruction from a large intramyocardial hydatid cyst, only a Grade III systolic murmur was heard. Unlike the high-velocity turbulent flow seen in fixed valvular stenosis, the cyst caused smooth, extrinsic, and relatively compliant compression, generating less turbulence and acoustic energy. Its intramyocardial location may have further limited sound transmission. As murmur intensity depends on flow dynamics, lesion geometry, and acoustic transmission, not just pressure gradients,^15^ severe RVOT obstruction may present with subtle findings, highlighting the importance of Doppler echocardiography for accurate detection.

Echocardiography is the first-line modality for diagnosing cardiac hydatid cysts, with >90% sensitivity.^5^ CT and MRI, which provide superior spatial resolution, precise anatomical localization, and characterization of cyst wall calcifications that are invaluable for surgical planning,^6^ are often unavailable and unaffordable in resource-limited settings. Therefore, echocardiography is essential for identifying characteristic features, such as the ‘cyst-in-cyst’ appearance, and for assessing the haemodynamic impact, including RVOT obstruction.^16^ A large systematic review (1210 cases) further supports its primary role, with TTE used in 84% of patients compared with 56.4% for CT and 34% for MRI.^17^ In this case, echocardiography reliably identified the characteristic ‘cyst-in-cyst’ appearance and severe RVOT obstruction. Due to the patient's limited financial resources, advanced imaging modalities such as CT and MRI were not utilized, and surgical decision-making relied exclusively on echocardiographic findings.

Serological tests like ELISA and IHA (sensitivity of 96.7% and specificity of 97.5% for ELISA and 86.7% and 95% for IHA) are more accurate than the traditional Casoni test in the diagnosis of hydatid disease, which has only 59.3% sensitivity and 60% specificity.^7,18,19^ Although the Casoni test was positive in this case, it can still be a useful supportive tool when combined with characteristic echocardiographic findings, especially in low-income settings.

The management of cardiac echinococcosis mandates a multidisciplinary approach combining definitive surgical excision and pharmacological therapy. Unlike asymptomatic abdominal cysts where a ‘watch-and-wait’ strategy may be employed, cardiac cysts warrant urgent surgical intervention due to the high risk of fatal complications, including anaphylactic shock, systemic or pulmonary embolism, and pericardial tamponade.^17,20^ In our patient, complete enucleation of the germinative membrane followed by capitonnage successfully relieved the RVOT obstruction, with postoperative gradients normalizing to 25/8 mmHg. This approach is consistent with the literature: Onursal *et al*.^21^ reported favourable outcomes in eight surgically treated cardiac echinococcosis cases, while Bayezid *et al*.^20^ described a case of interventricular septal hydatid cyst complicated by pulmonary embolism, emphasizing the critical importance of timely surgical intervention.

Pharmacological management is critical to reduce cyst viability preoperatively and prevent recurrence post-operatively. Albendazole, the first-line agent, acts by inhibiting microtubule formation in the parasite, thereby impairing glucose uptake and leading to parasite death, while mebendazole, another benzimidazole with a similar mechanism but lower systemic bioavailability, and praziquantel, a quinoline, derivative, serve as alternative options.^22^ In our patient, albendazole was administered at 400 mg orally twice daily, initiated 1 month preoperatively and continued for 6 months post-operatively, consistent with current recommendations.^17^

The post-operative echocardiographic evaluation demonstrated complete cyst excision and resolution of RVOT obstruction, along with improvement in the patient's symptoms, supports the literature, which indicates a favourable prognosis when early diagnosis is combined with medical and surgical treatment, resulting in low recurrence rates.^3,20^

Echinococcosis remains highly endemic in many low-income regions, including Afghanistan, driven by poor sanitation, inadequate hygiene, limited access to healthcare, low health literacy, and close contact with infected canids.^1^ Given these conditions, Afghanistan likely has a higher prevalence of cardiac hydatid disease than currently documented. Although non-cardiac hydatid disease has been reported locally, no published cases of cardiac hydatid cysts exist to date. This gap highlights the need for further research, as underdiagnosis and underreporting may conceal the actual burden of disease.

## Conclusion

This case highlights the rare presentation of an isolated interventricular septal hydatid cyst, which manifested with syncope. It underscores the critical importance of early diagnosis through echocardiography along with serologic tests in resource-limited settings where advanced imaging modalities are not available to prevent life-threatening complications. Cardiac hydatid cystsespecially those involving the interventricular septum—are rare, yet they should always be considered in patients from endemic regions, even when obvious cardiac symptoms are absent. Optimal management relies on timely surgical excision combined with anti-parasitic therapy. Effective outcomes require strong multidisciplinary collaboration among cardiologists, infectious disease specialists, and cardiac surgeons to ensure accurate diagnosis and comprehensive treatment.

## Data Availability

The data supporting the findings of this study are included in this paper.

## Lead author biography

Abdul Wahed Sidiqi, an MD graduate and lecturer at Kabul University of Medical Sciences, is a leading figure in Afghanistan’s cardiology. A Fellow of the European Society of Cardiology and Head of the Afghanistan Cardiovascular Association, he founded the Ariana Medical Complex and advanced cardiology practice and echocardiography training. After training in the Netherlands and South Korea, he introduced BLS and ACLS in Afghanistan. He has published several articles, including “Global Rounds Afghanistan: A Critical Overview of Cardiovascular Medicine” in Circulation, and has served as a speaker at numerous international cardiology conferences, earning the Best Abstract Award at the ACHD Congress in Madrid (2026).

## Notes

**Consent:** The patient provided written informed consent for the publication of this case report and any accompanying images and clinical information. The authors confirm that consent was obtained before submission and that publication complies with the Committee on Publication Ethics (COPE) guidance regarding patient consent for case reports.

## References

[1] World Health Organization (WHO) . WHO. 2021. Echinococcosis: https://www.who.int/news-room/fact-sheets/detail/echinococcosis

[2] Markanday K, Rafath S, Radhakrishnan B. Cardiac echinococcosis. APIK J Intern Med [Internet] 2024;14:1–3.

[3] Soufiani A, El-Mhadi S, Chraibi H, Agoumy Z, Fehri ZF, Es-Sebbani S, et al The silent invader: a case of intrapericardial hydatid cyst with exceptional pulmonary artery involvement. Oxford Med Case Reports 2023;2023:340–342. 10.1093/omcr/omad099 PMC10530307 37771683

[4] Shanmugam K, U K, Sadiq AM, Govind SC, Raj V, Raut BK. Incidental left ventricular hydatid cyst in a patient with chest pain: a case report. Indian J Clin Cardiol 2024;5:367–371.

[5] Mir H, McClure A, Thampinathan B, Chow C, Cusimano RJ, Bogoch II, et al Echocardiographic features of cardiac echinococcal infection. Case 2021;5:26–32. 33644510 10.1016/j.case.2020.10.002 PMC7887517

[6] Petik B, Hazirolan T, Uysal G, Erturk SM. Cardiac hydatid cysts computed tomography and magnetic resonance imaging findings of the 5 cases. J Comput Assist Tomogr 2015;39:816–819. 26196344 10.1097/RCT.0000000000000284

[7] Erganis S, Sarzhanov F, Doğruman F, Kayhan A. Comparison of methods in the serologic diagnosis of cystic echinococcosis. Acta Parasitologica 2024;69:1122–1131. 38551763 10.1007/s11686-024-00840-z PMC11182860

[8] Moro P, Schantz PM. Echinococcosis: a review. Int J Infect Dis 2009;13:125–133. 18938096 10.1016/j.ijid.2008.03.037

[9] Wegner B, Meel R, Nell T, Nqwata L, Wong M. Hydatid disease of the interventricular septum: echocardiographic and computed tomography findings. South African J Radiol 2020;24:1–4. 10.4102/sajr.v24i1.1986 PMC7756595 33391841

[10] Shojaei E, Yassin Z, Rezahosseini O. Cardiac hydatid cyst: a case report. Iran J Public Health 2016;45:1507–1510. 28028503 PMC5182260

[11] Yan F, Huo Q, Abudureheman M, Qiao J, Ma SF, Wen H. Surgical treatment and outcome of cardiac cystic echinococcosis. Eur J Cardio-thoracic Surg [Internet] 2014;47:1053–1058. 10.1093/ejcts/ezu323 25193952

[12] Yaman M, Hakan A, Arslan U, Ozturk H, Aksakal A. Case report A giant cardiac hydatid cyst presenting with chest pain and ventricular tachycardia in a pregnant woman undergoing cesarean section. Indian Heart J 2016;68:S118–S120. 27751261 10.1016/j.ihj.2016.04.011 PMC5067732

[13] Zeng YH, Calderone A, Rousseau-saine N, Couture J, Elmi-sarabi M, Aldred MP, et al Systematic review/meta-analysis right ventricular out flow tract obstruction in adults: a systematic review and meta-analysis. CJC open 2021;3:1153–1168. 34746729 10.1016/j.cjco.2021.03.011 PMC8551422

[14] Sabzi F, Faraji R. Hydatid cyst of the interventricular septum causing complete heart block and postoperative ventricular septal defect. Indian J Crit Care Med 2014;18:473–475. 25097364 10.4103/0972-5229.136080 PMC4118517

[15] McGee S . McGees Evidence-Based Physical Diagnosis. 6th ed. Philadelphia, PA: Elsevier; 2026. p439.

[16] Diaz FJ, Hospital H. Diagnostic value of two-dimensional echocardiography in cardiac hydatid disease. Eur Heart J 1991;12:1300–1307. 1778196 10.1093/eurheartj/12.12.1300

[17] Bumann S, Kuenzli E, Lissandrin R, Brunetti E, Goblirsch S, Henning L, et al Cardiac cystic echinococcosis—a systematic review and analysis of the literature. PLoS Negl Trop Dis 2024;18:1–22. 10.1371/journal.pntd.0012183 PMC11139302 38814859

[18] Kumar R, Naik MI, Ahmad J, Ahmad B. Comparison of Casoni’s intradermal test with enzyme linked immunosorbent assay in the diagnosis of human hydatid disease 2013.

[19] El-Shazly AM, Saad RM, Belal US, Sakr T, Zakae HA. Evaluation of ELISA and IHAT in serological diagnosis of proven cases of human hydatidosis. J Egypt Soc Parasitol 2010;40:531–538. 21246959

[20] Bayezid O, Ocal A, Isik O, Okay T, Yakut C. A case of cardiac hydatid cyst localized on the interventricular septum and causing pulmonary emboli. J Cardiovasc Surg (Torino) 1991;32:324–326. 2055928

[21] Onursal E, ElmacI TT, TirelI E, Dindar A, AtIlgan D, Özcan M. Surgical treatment of cardiac echinococcosis: report of eight cases. Surg Today 2001;31:325–330. 11321342 10.1007/s005950170153

[22] Soleymani N, Sadr S, Santucciu C, Rahdar A, Masala G. Evaluation of the in-vitro effects of albendazole, mebendazole, and praziquantel nanocapsules against protoscolices of hydatid cyst. Pathogens (Basel, Switzerland) 2024;13:790. 39338980 10.3390/pathogens13090790 PMC11435210

